# Base-pair resolution DNA methylome of the EBV-positive Endemic Burkitt lymphoma cell line DAUDI determined by SOLiD bisulfite-sequencing

**DOI:** 10.1038/leu.2013.4

**Published:** 2013-02-01

**Authors:** B Kreck, J Richter, O Ammerpohl, M Barann, D Esser, B S Petersen, I Vater, E M Murga Penas, C A Bormann Chung, S Seisenberger, V Lee Boyd, S Smallwood, H G Drexler, R A F MacLeod, M Hummel, F Krueger, R Häsler, S Schreiber, P Rosenstiel, A Franke, R Siebert

**Affiliations:** 1 Institute of Clinical Molecular Biology, Christian-Albrechts-University of Kiel, Kiel, Germany; 2 Institute of Human Genetics, Christian-Albrechts-University of Kiel and University Hospital Schleswig-Holstein, Campus Kiel, Kiel, Germany; 3 Life Technologies, Foster City, CA, USA; 4 The Babraham Institute, Cambridge, UK; 5 Department of Human and Animal Cell Cultures, German Collection of Microorganisms and Cell Cultures, Braunschweig, Germany; 6 Institute of Pathology, Charité—University Medicine Berlin, Berlin, Germany; 7 Department of General Internal Medicine, University Hospital Schleswig-Holstein, Campus Kiel, Kiel, Germany

The Burkitt translocation t(8;14), first identified in the 1970s in biopsies and cell lines from Burkitt lymphoma (BL),^1, 2^ and its variants juxtapose the *MYC* oncogene to one of the immunoglobulin (*IG*) loci.^3^ Nowadays, it is assumed that (nearly) all BL carry an *IG-MYC* translocation, rendering this somatic mutation a diagnostic marker for all three subtypes of BL (endemic, sporadic and immunodeficiency-related BL).

In contrast to many other lymphomas, BL show a quite simple karyotype, that is, with few if any secondary chromosomal changes in addition to the *IG-MYC* translocation.^4^ Although there is evidence for some few recurrent secondary genetic changes the number of epigenetic alterations in BL as compared with normal B-cell subsets seems to outnumber the genetic changes by far.^5, 6, 7^ Indeed, along with others, we have identified several hundred genes showing *de novo* DNA methylation in aggressive B-cell lymphoma, including BL as compared with normal B-cell subsets.^8, 9, 10^ Nevertheless, the mentioned DNA methylation studies focused on a maximum of probably 10% (by HELP assays) of the CpGs of the genome, and were biased toward promoter regions and CpG islands and did not systematically analyze non-CpG methylation.^8, 9, 10, 11^ Therefore, we here aimed at generating a complete DNA methylome of a BL, allowing for unbiased analyses of all cytosines in the genome.

To this end, we chose the archetypal DAUDI cell line, established from an endemic BL (eBL) that was derived from a 16-year-old African male patient in 1967.^12, 13^ We selected this cell line as it has been pivotal for the identification of t(8;14), still carries a simple karyotype despite being many years in culture and because it shows the prototypic features of eBL. Moreover, considering the strong association of eBL with Epstein–Barr virus (EBV) infection, the EBV-positive DAUDI cell line offers the opportunity for a direct comparison of its lymphoma and EBV methylomes.

To obtain a base-pair resolution DNA methylome of a prototypic eBL, we subjected DNA of the DAUDI cell line to full bisulfite-sequencing (BS-seq) using the SOLiD two-base encoding (colorspace) approach (for details, see Supplementary Information). Two bisulfite-converted SOLiD fragment libraries were constructed. Briefly, 15 μg of genomic DNA were sheared to approximately 125 bp. After end-repair of the DNA fragments, methyl-P1 and -P2 adaptors were ligated. The DNA was then size selected and nick translated with a modified deoxyribonucleoside triphosphate (dNTP) mix containing methyl-deoxycytidinetriphosphate (dCTP) instead of regular dCTPs. Bisulfite conversion was carried out in solution and recovered DNA fragments were PCR amplified using eight cycles. The bisulfite-converted fragment library was clonally amplified on SOLiD P1 beads using emulsion PCR. Templated (P2 positive) beads were then enriched and deposited on a slide for sequencing. Technical details on the genomic characterization of the cell line including karyotyping, single-nucleotide polymorphism (SNP) array analysis and exome sequencing as well as on control DNA methylation analyses using Illumina 450K BeadArray analysis (Illumina, San Diego, CA, USA), limited BS-seq using Illumina HiSeq2000 technology as well as Luminometric methylation assay (LUMA) are provided in the Supplementary Information.

By karyotyping and SNP-array analysis, we confirmed that the cells under study show the typical features of BL, including the t(8;14) plus a few secondary chromosomal changes. Exome sequencing followed by filtering for known SNPs revealed a total of 2313 non-synonymous mutations (Supplementary Table 1a). Owing to the lack of a germline control from the patient from which the DAUDI cell line has been established, it is not possible to reliably differentiate somatic (lymphoma-associated) mutations from germline variants. Despite this limitation, exome sequencing in line with recent reports,^5, 6, 7^ identified inactivating mutations in the *ID3* gene, which have been shown to co-operate with MYC activation in the pathogenesis of BL.^5, 6, 7, 14^ In addition, Sanger sequencing confirmed sequence variants in the genes *B2M, TET2* and *KIT* (Supplementary Table 1b).

We aligned 79.9 Gb of BS-seq of the SOLiD platform.^15^ These were compared with 7.8 Gb aligned BS-seq of the HiSeq 2000 platform and the results to the DNA methylation levels determined by HumanMethylation450 BeadChip analysis (Supplementary Figure S1). We observed high correlation of the SOLiD data with both the sequence-based HiSeq 2000 (Pearson *r*=0.86; Supplementary Figure S2) and the array-based (Pearson *r*=0.96; Supplementary Figure S3) methylation levels. This led us to focus our further analyses on the most extensive data set derived from SOLiD BS-seq.

In total, 91.1% of all CpG sites and 90.2% of all non-CpG sites of the genome were covered by at least five SOLiD reads (Supplementary Figure S4). On the genome-wide level, 68.99% cytosines in CpG dinucleotides were methylated, which is in line with previous pyrosequencing-based determinations using LUMA. In contrast, the 450K BeadArray shows a mean methylation level of 59.24%, which is mostly due to the selection bias of the array loci, which are predominantly located within regions upstream of genes. We observed a mean CpG methylation level of 65.79% in LINEs and 78.84% in SINEs (Supplementary Figure S5). BS-seq shows the DNA methylation patterns on the forward and reverse strand to be comparably established (Pearson *r*=0.90).

Considering the recent description of non-CpG methylation in embryonic stem cells (ESCs), and the fact that the *MYC* oncogene deregulated in BL is also one of the four factors used to induce a stem cell-like phenotype in differentiated cells,^16^ we analyzed the level of non-CpG methylation. The genome-wide fraction of methylated cytosines in a non-CpG context does not exceed the respective threshold of 0.003 given by the unmethylated lambda control DNA that was tested in parallel.^11^ Moreover, we confirmed absence of non-CpG methylation at hallmark sites described in ESC^11^ by bisulfite pyrosequencing (Supplementary Figure S6). Despite this overall low frequency of non-CpG methylation, we could identify a remarkable 6.7-fold enrichment of methylated non-CpG sites within genes (*P*<2.2 × 10^−16^; Figure 1b). Such non-CpG methylation might be linked to transcriptional activity (Supplementary Figure S7).

We next determined the sequence-based methylation status of 969 genes recently shown by us to exhibit *de novo* promoter hypermethylation in mature aggressive B-cell lymphoma (including BL) as compared with normal B cells.^8^ We could confirm that in DAUDI cells 91.21% of these genes have a DNA methylation level ⩾60% in their promoter region and lack transcription. As compared with all other RefSeq genes, the mean CpG methylation level within promoter regions of the 969 genes was significantly higher (84% vs 41% Supplementary Tables S2).

Gene expression analyses confirmed that DAUDI cells show the typical signature of molecular BL.^17^ Correlating methylation and expression patterns in our data revealed that significant presence of transcripts is associated with absence of DNA methylation particular at and closely around the transcription start site (TSS). In contrast, DNA methylation exactly at the TSS correlates with lack of transcription (Figure 2). Although the group of non-expressed genes showed an overall high mean DNA methylation level across the whole gene with highest methylation levels in exons, genomic regions comprising expressed genes were characterized by particular high methylation levels in the first intron. Moreover, the patterns of both expressed and non-expressed genes were characterized by sharp transitions of methylation levels at exon–intron borders (Figure 2). Overall, when compared with transcriptional activity the patterns of DNA methylation across different parts of genes were similar to those recently determined in non-neoplastic tissues including blood.^18^

Finally, we studied the DNA methylation of the mitochondrial and EBV genomes of DAUDI cells.^19, 20, 21^ We estimated 80 EBV and 370 mitochondrial copies per DAUDI cell based on coverage analyses, which is in accordance with previous studies.^22^ Although mitochondrial DNA is mostly unmethylated (mean methylation 6.43% Figure 1a), CpG methylation in the human and EBV genome is comparably distributed, although the EBV genome exhibits hardly any fully methylated sites (Figure 1a). Overall, EBV shows a high level of DNA methylation (mean methylation 80.18%), as it was previously shown for BL cell lines.^23^ Nevertheless, DNA methylation within the EBV genome correlates with expression only at high transcript levels (fragments per kilobase of exon model per million mapped (FPKM)⩾15) (Supplementary Figure S8).

In summary, we have characterized the nuclear DNA methylome of an eBL along with its mitochondrial and EBV methylome using colorspace BS-seq. We unravel significant differences between the different sub-methylomes and moreover show that gene transcription is associated with complex patterns of methylation, extending beyond simple promoter and CpG methylation. As the DAUDI cell line has been used over decades in many laboratories in the world, the obtained methylome data might serve as a ‘reference epigenome' for future studies.

Data availability: methylome data are available at ftp://134.245.63.215/export/home/daudi' (login: daudi; password: daudismethylome2012).

## Figures and Tables

**Figure 1:**
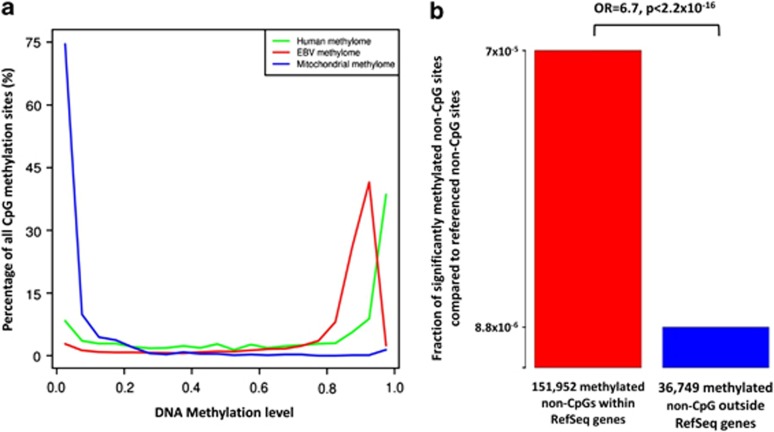
Distribution of DNA methylation in DAUDI cells. (**a**) The graphs show genome-wide distributions of CpG methylation of the human nuclear, EBV and mitochondrial genomes. The y axis indicates DNA methylation levels assessed by SOLiD BS-seq. Green: human nuclear CpG methylation; red: EBV CpG methylation; blue: mitochondrial CpG methylation. (**b**) Significantly methylated non-CpG sites of DAUDI within RefSeq genes (comprising 424 969 306 non-CpGs) are 6.7-fold enriched compared with those outside of RefSeq genes (comprising 689 750 660 non-CpGs). Red: fraction of significantly methylated non-CpGs of DAUDI within RefSeq genes; blue: fraction of significantly methylated non-CpGs of DAUDI outside of RefSeq genes. OR: odds ratio.

**Figure 2:**
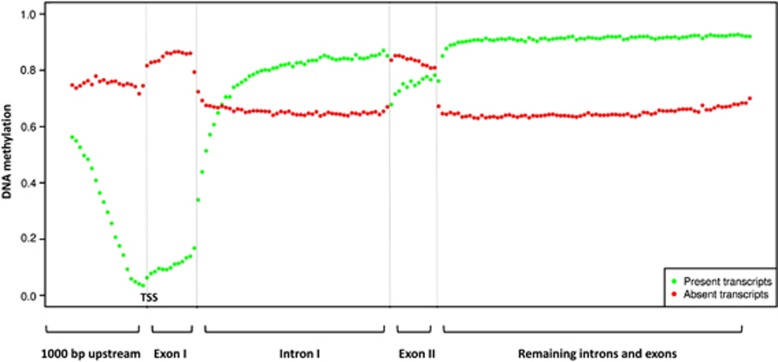
Correlation of DNA methylation levels and transcriptional states. CpG methylation levels were averaged for annotated RefSeq gene regions and transcripts are clustered by their expression level in present (*n*=7662 transcripts) and absent (*n*=5429 transcripts) calls. A strong dependency of the location of CpGs related to their distance to the TSS and the transcript expression level can be observed. Green: average methylation pattern for present transcripts; red: average methylation pattern for absent transcripts.

## Notes

OA and RS received research support by Illumina on BeadChip Analyses. CABC is and VLB used to be employed by Life Technologies. The remaining authors declare no conflict of interest.
