# Supplementary material for: Base-pair resolution DNA methylome of the EBV-positive Endemic Burkitt lymphoma cell line DAUDI determined by SOLiD bisulfite-sequencing
Source: Leukemia. 2013 Feb 1;27(8):1751–3. doi: 10.1038/leu.2013.4 (PMC3740476; doi:10.1038/leu.2013.4)
Supplement: Supplementary Information [file leu20134x1.doc]

**SUPPLEMENTARY INFORMATION**

**Base-Pair-Resolution DNA Methylome of the EBV-positive Endemic Burkitt Lymphoma Cell Line DAUDI determined by SOLiD™ Bisulfite-Sequencing**

**Running title**: Bisulfite-sequenced Methylome of Burkitt Lymphoma

B Kreck^1^, J Richter^2^, O Ammerpohl^2^, M Barann^1^, D Esser^1^, BS Petersen^1^, I Vater^2^, EM Murga Penas^2^, CA Bormann Chung^3^, S Seisenberger^4^, V Lee Boyd^3^, S Smallwood^4^, HG Drexler^5^, RAF MacLeod^5^, M Hummel^6^, F Krueger^4^, R Häsler^1^, S Schreiber^1,7^, P Rosenstiel^1^, A Franke^1*^and R Siebert^2*^

^1^Institute of Clinical Molecular Biology, Christian-Albrechts-University of Kiel, Kiel, Germany;

^2^Institute of Human Genetics, Christian-Albrechts-University of Kiel & University Hospital Schleswig-Holstein, Campus Kiel, Kiel, Germany;

^3^Life Technologies, Foster City, CA, United States of America;

^4^The Babraham Institute, Cambridge, United Kingdom;

^5^Department of Human and Animal Cell Cultures, German Collection of Microorganisms and Cell Cultures, Braunschweig, Germany;

^6^Institute of Pathology, Charité – University Medicine Berlin, Germany;

^7^Department of General Internal Medicine, University Hospital Schleswig-Holstein, Campus Kiel, Kiel, Germany

*AF and RS share senior authorship

**SUPPLEMENTARY MATERIAL AND METHODS**

**Genomic characterization of DAUDI cells**

DAUDI cells (ACC-078) and DNA were provided by the “Deutsche Sammlung von Mikroorganismen und Zellkulturen*”* (DSMZ). Chromosomal R-banding analysis and Genome-Wide Human SNP Array 6.0 (Affymetrix, Santa Clara, CA, USA) analysis were performed according to standard methods. Whole exome capture and sequencing were carried out using Illumina’s TruSeq Exome Enrichment Kit (Illumina, San Diego, CA, USA). A subset of mutations identified by exome sequencing was verified by Sanger Sequencing.

The cell line DAUDI has been established in 1967 from an endemic BL (eBL) presenting in the left orbita of a 16-year-old African boy.^1^ The cell line is positive for EBV (HHV-4) but lacks expression of immediate-early protein BZLF-1 and lately expressed capsid protein. By PCR, the cell line is negative for HBV, HCV, HHV-8, HIV, HTLV-I/II and SMRV. The immunophenotype has been determined as CD3 -, CD10 +, CD19 +, CD20 +, CD37 +, CD38 +, cyCD79a +, CD80 +, CD138 -, HLA-DR +, sm/cyIgM +, sm/cyIgG -, sm/cykappa +, sm/cylambda – (data available from DSMZ at www.dsmz.de).

Cytogenetic analysis using chromosomal R-banding revealed a karyotype 46,XY,t(8;14)(q24;q32). These findings are in line with those from Multicolor Fluorescence In Situ Hybridization (M-FISH) of the DAUDI cell line and showing that it has remained karyotypically stable along decades of continuous cultivation (EMMP *et al.*, manuscript in preparation).

The Genome-Wide Human SNP Array 6.0 was performed according to manufacturer’s protocol (Affymetrix, Santa Clara, CA) using the Fluidics Station 450 and the GeneChip Scanner 3000 (Affymetrix, Santa Clara, CA). The Birdseed v2 algorithm was used to genotype tumour samples. Copy number analysis, Loss of heterozygosity (LOH) analysis and segmentation was calculated using Genotyping Console software version 3.0 (Affymetrix, Santa Clara, CA). Segments with significant imbalances were considered as copy number aberration only if they consisted of at least 20 sequential probes, comprised a minimal size of 100 kb, and mapped outside known copy number polymorphisms. Data analysis revealed two gains and four chromosomal losses: arr 4q13.3(75,205,069-75,765,823)x1,5p11.1(46,361,933-49,591,883)x1,7q31.32q31.33(121,093,529-123,683,639)x1, 8q24.21q24.3(128,682,912-146,268,947)x3,14q32.31q32.33(101,614,613-105,400,262)x4,15q12q21.1(25,151,945-45,290,444)x1 (NCBI36/hg 18)

**DNA methylation profiling using Bisulfite-Sequencing (BS-seq)**

We performed genome-wide BS-seq on the SOLiD™ (Life Technologies, Carlsbad, CA) and the HiSeq 2000 platform.

For the prior, two bisulfite-converted SOLiD™ fragment libraries were constructed as described previously.^2,3^ Briefly, 15 µg of genomic DNA were sheared to approximately 125 bp using a Covaris S2 system (Life Technologies, Carlsbad, CA, USA). After end-repair of the DNA fragments, methyl-P1 and P2 adaptors were ligated (for details on methyl-P1 and P2 see Ranade *et al.*^3^). The DNA was then size selected on an agarose gel and nick-translated with a modified dNTP Mix containing methyl-dCTPs instead of regular dCTPs in order to protect the adaptor sequences during bisulfite conversion. Bisulfite conversion was carried out in solution as described previously^3^ and recovered DNA fragments were PCR amplified using 8 cycles. The bisulfite converted fragment library was clonally amplified on SOLiD P1 beads using emulsion PCR. Templated (P2 positive) beads were then enriched and deposited on a slide for sequencing.

For the HiSeq 2000 analyses, genomic DNA was sonicated using the Diagenode Bioruptor (Diagenode, Denville, NJ) to a final size distribution ranging from 100 bp to 800 bp. Libraries were prepared from the sonicated DNA using the NEBNext Sample Prep Master Mix Set 1 (New England Biolabs, Ipswich, MA) according to the manufacturers’ instructions. Illumina’s Early Access Methylation Adapter Oligo was used for the ligation. The adapter-ligated DNA was treated with sodium bisulfite using the Imprint DNA Modification Kit (Sigma, St. Louis, MO) according to the manufacturers’ instructions. The bisulfite-treated product was amplified with 16 cycles using a uracil stalling-free polymerase (Agilent, Santa Clara, CA, USA) followed by size selection on a gel (200 bp – 250 bp) and purification with the Qiagen Gel Extraction Kit (Qiagen, Hilden, Germany).

Sequencing using SOLiD™ v4.0 chemistry according to manufacturer’s instructions yielding 50 bp reads, which were analyzed with B-SOLANA.^4,5^ For the HiSeq 2000 analyses, V3 chemistry was used according to manufacturer’s instructions yielding 100 bp reads, which were analyzed with Bismark.^5,6^ A comparison of the different bisulfite-sequencing techniques has been recently published. ^5^

**DNA methylation profiling using universal BeadArrays**

DNA of DAUDI cells was subjected to 450K BeadArray-analysis. Bisulfite conversion was performed using the Zymo EZ DNA Methylation Kit (Zymo Research, Orange, CA, USA) according to the manufacturer’s instruction. Subsequent analysis steps were performed according to the manufacturer’s protocol measuring DNA methylation at >485,000 CpG sites selected from more than 21,000 genes in parallel. Hybridization signals were analyzed using GenomeStudio software (default settings; GenomeStudio ver. 2011.1, Methylation Analysis Module ver. 1.9.0; Illumina Inc) and internal controls for normalization.

**Gene expression analyses**

We combined expression data from the U133A gene chip (Affymetrix, Santa Clara, CA) and RNA-sequencing (RNA-seq) using the SOLiD™ platform (Life Technologies, Carlsbad, CA). The RNA-seq library was prepared by a modified Whole Transcriptome Analysis Kit (WTAK) (Life Technologies, Carlsbad, CA) and analyzed as previously described,^7^ with the modification that we used TopHat^8^ to carry out alignments.

U133A raw data was analyzed using the *panp* package of the R statistical software (Peter Warren, panp R package version 1.20.1.) and the Affymetrix Microarray Analysis Suite version 5.0 (MAS).^9^

The paired-end RNA-seq library was prepared using the RiboMinus™ Eukaryote Isolation Kit and the RiboMinus™ Concentration Kit (Life Technologies, Carlsbad, CA). Subsequently, the SOLiD™ Whole Transcriptome Analysis Kit (WTAK) was performed and sequencing was carried out at 50 bp in the forward and 35 bp in the reverse direction using SOLiD™ v4.0 chemistry according to manufacturer’s instructions.

A combined set of results was generated in the following manner. Present transcripts are the intersection of U133A-determined present calls and RNA-seq calls consisting of a FPKM value >0.01. However, transcripts including an absent assignment by the U133A assay and a FPKM value ≤0.01 were considered as an absent call. In total, we could assess 7662 present calls and 5429 absent calls.

Expression analyses of EBV are restricted to RNA-seq data, due to missing EBV-annotations on the U133A-chip. We raise the FPKM threshold for transcripts within the EBV genome from 0.01 to 1 estimated by the number of ≈100 copies (compared to ≈80 copies assessed by coverage analyses) of virus particles per cell.^10^

**Whole Exome Sequencing**

We performed whole exome capture using Illumina’s TruSeq Exome Enrichment Kit and sequencing of 2x100 bp paired-end reads was performed on one quarter lane of an Illumina HiSeq2000. Reads were mapped against the human reference genome build hg18 using BWA,^11^ followed by the removal of PCR duplicates with Picard (http://picard.sourceforge.net). Variant calling was performed with SAMtools mpileup and GATK,^12,13^ for SNP annotation and filtering we applied our own in-house tool *snp*Acts. InDels were annotated using ANNOVAR.^14^

A total of 62,578 on target SNPs were filtered against 8 exomes of healthy individuals, allowing for a maximum frequency of 1% in the 1000 genomes project and keeping only non-synonymous and splice-site SNPs that were not present in dbSNP130 resulting in 2,313 SNP after filtering.

**Sanger Sequencing**

Selected SNVs detected by exome sequencing were verified by Sanger Sequencing on an ABI Sequencer 3100 (Applied Biosystems).

Primer sequences used for validation of potentially protein changing mutations:

| **Gene** | **Primer name** | **Sequence 5´-3´** |
| --- | --- | --- |
| *B2M* | B2M_FP | TCCCTCTCTCTAACCTGGCAC |
|  | B2M_RP | ACTTGGAGAAGGGAAGTCACG |
| *TET2* | TET2_FP | TGCATGCAAAATACAGGTTTC |
|  | TET2_RP | CAGCTTGCAGGTGGATTCTC |
| *ID3* | ID3_FP | TCCAGGCAGGCTCTATAAGTG |
|  | ID3_RP | CCGAGTGAGTGGCAATTTTT |
| *KIT* | KIT_FP | CACAGACCCAGAAGTGACCA |
|  | KIT_RP | TACCTGGCCTCACTTTCAGG |

**Identification and analyses of significant non-CpG methylation sites**

Potential methylcytosines in a non-CpG context were detected as described by Lister *et al.*.^15^ Hereby, the binomial distribution was used to exclude false positive non-CpG methylation sites arisen by incomplete bisulfite conversion. Furthermore, we corrected for potential non-CpG sites including a mutation (N to G) in their adjacent base (+1). Although the genome-wide amount of methylated cytosines in non-CpG context does not exceed the expected threshold, given by an estimation based on spiked-in lambda phage, we were able to identify a local enrichment of non-CpG methylcytosines within gene regions (Figure 1b). In detail, we observed 9533 RefSeq genes containing significant non-CpG sites. The ratio of these present and absent transcripts is slightly enriched compared to the genome-wide level (OR=1.11). However there is no significant difference between absent and present transcripts in terms of the ratio of significant and existing non-CpG sites within RefSeq genes (Table S7).

**Bisulfite Pyrosequencing**

Bisulfite pyrosequencing of two regions identified by Lister et al., to carry non-CpG methylation in differentiated and stem cells were pyrosequenced.^15^ Bisulfite pyrosequencing was carried out as described by Lamprecht *et al.*.^16^ Briefly, genomic DNA was bisulfite converted using the EpiTect Bisulfite Conversion Kit (Qiagen). In a subsequent PCR amplification locus-specific primers were used with one primer biotinylated at the 5´ end (primer sequences are shown below). Amplification was verified by agarose gel electrophoresis. Using the VacuumPrep Tool (Biotage, Uppsala, Schweden) single strands were prepared followed by a denaturation step at 85°C for two minutes and final sequencing primer hybridization. Pyrosequencing was performed using the Pyrosequencer ID and the DNA methylation analysis software Pyro Q-CpG 1.0.9 (Biotage), which was also used to evaluate the ratio T:C (mC:C) at the CpG sites analyzed. All assays were optimized and validated using commercially available completely methylated DNA (Millipore) and pooled DNA isolated from peripheral blood of 10 healthy male and female controls, respectively.

Primer sequences used for bisulfite pyrosequencing:

| **Primer name** | **5´-3´sequence** | **5´modification** |
| --- | --- | --- |
| methyl_chr1_FP | AAATTTGGTTTTTTTATATGG |  |
| methyl_chr1_RP | CTAAAACCTCTTAAACTTTTATCA | Biotin |
| methyl_chr1_seq | GGTTTTTTTATATGGTTA |  |
| methyl_chr10_FP | GATGGGTGATTTTTTAGA |  |
| methyl_chr10_RP | ACATTTCCTACAATTTCAA | Biotin |
| methyl_chr10_seqa | TGGGTGATTTTTTAGAGTT |  |
| methyl_chr10_seqb | GATTTGTGGAAGATAGA |  |

**Luminometric Methylation Assay (LUMA)**

To analyze global genomic DNA methylation, LUMA was performed as previously described.^17^

**SUPPLEMENTARY RESULTS**

**Supplemental Table 1: SNP filtering of Exome sequencing data (a) and potentially protein changing mutations detected by Exome sequencing and validated by Sanger Sequencing (b)**.

Supplementary Table 1a

| SNPs on target | 62,578 |
| --- | --- |
|  | ↓ |
| SNPs not in healthy controls^*^ | 25,752 |
|  | ↓ |
| SNPs involving a frequency in 1000 genomes pilot of max. 1% | 18,822 |
|  | ↓ |
| SNPs not in dbSNP130 | 10,650 |
|  | ↓ |
| non-synonymous SNPs^**^ | **2,313** |

^*^Healthy controls are taken from published datasets^18,19^, whereas we solely used the HapMap probes published by Ng *et al..*^19^ Additionally, we included exome data generated in-house. ^**^Due to lack of germline control from the patient from which the DAUDI cell line has been established it is not possible to differentiate somatic (lymphoma-associated) mutations from germline variants.

**Supplementary Table 1b**

| **Gene** | **chr** | **position** | **ref/ alt allele** | **aa consequence** | **note** |
| --- | --- | --- | --- | --- | --- |
| *B2M* | 15 | 42791039 | G/C | p.Met1Ile | Confirmed |
| *TET2* | 4 | 106377217 | C/T | p.Ser911Leu | Confirmed |
| *ID3* | 1 | 23758264 | G/A | p.Leu54Val | Confirmed |
|  | 1 | 23758345 | G/C | p.Gln81* | Confirmed |
| *KIT* | 4 | 55259372 | C/T | p.Ala67Ser | Confirmed |

**Supplemental Tables S2: Hypermethylation in mature aggressive B-cell lymphoma.** Comparison of genome-wide DNA methylation within RefSeq genes (Table S2a) and hypermethylated genes associated with mature aggressive B-cell lymphoma including BL (Table S2b).

Supplemental Table S2a

| **RefSeq genes** | **1000 bp upstream** | **First exon** | **First intron** | **Second exon** | **Second exon to last exon** |
| --- | --- | --- | --- | --- | --- |
| Mean methylation (watson strand) | 0.40 | 0.40 | 0.70 | 0.80 | 0.78 |
| Mean methylation (crick strand) | 0.41 | 0.39 | 0.69 | 0.81 | 0.78 |

Supplemental Table S2b

| **Genes de novo methylated in mature aggressive B-cell lymphoma**^20^ | **1000 bp upstream** | **First exon** | **First intron** | **Second exon** | **Second exon to last exon** |
| --- | --- | --- | --- | --- | --- |
| Mean methylation (watson strand) | 0.84 | 0.90 | 0.63 | 0.81 | 0.60 |
| Mean methylation (crick strand) | 0.84 | 0.90 | 0.66 | 0.84 | 0.63 |

**Supplemental Figure S1: Comparison of four HumanMethylation450 BeadArray replicates.** Scatter plots depict the comparison of all four replicates of 450K runs of DAUDI cells. All of them show high correlations among each other r≥0.99.

**Supplemental Figure S2: Comparison of SOLiD™ and Illumina BS-seq.** The histogram depicts the differences (SOLiD™ BS-seq – Illumina BS-seq) in methylation levels ((methylated reads)/(unmethylated reads + methylated reads)) for CpGs with coverage of at least 5 reads. This 5x coverage was applied as a cut-off as it is in the range of those applied in recently published whole genome BS-seq analyses with SOLiD and HiSeq technologies.^21,22^ Values close to 0.0 indicate that equal methylation levels were inferred by both methods. Both approaches show comparable genome-wide DNA methylation levels r=0.86.

**
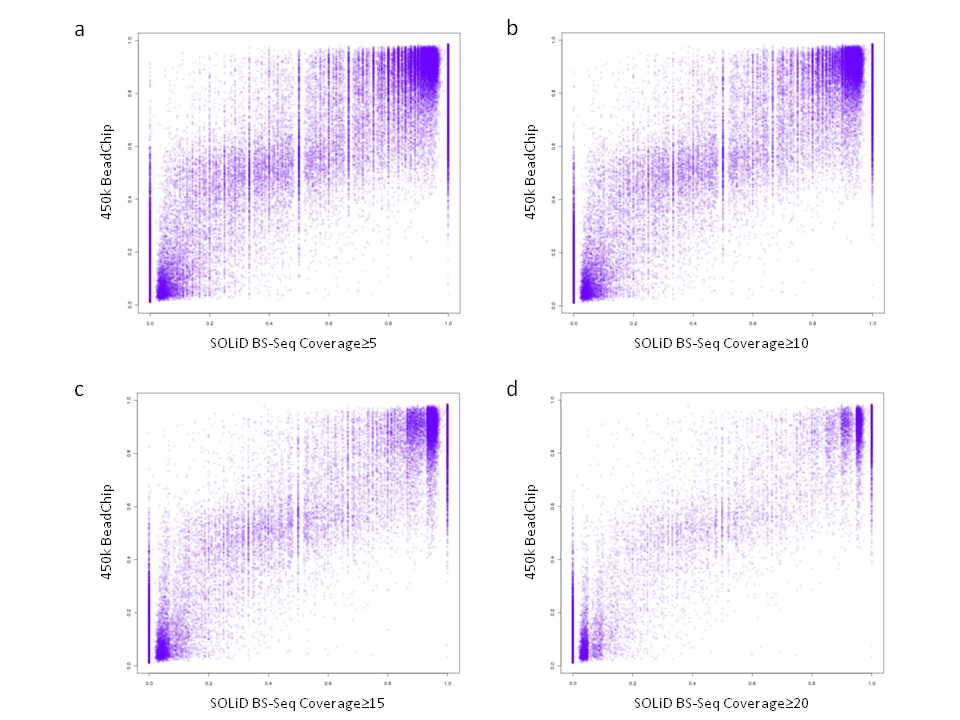
**

**Supplemental Figure S3: Comparison of SOLiD™ BS-seq and HumanMethylation450 BeadArray data.** The scatter plots depict the comparison of SOLiD™ BS-seq and 450k methylation levels. Higher read coverage generally results in better correlation. (a) includes SOLiD™ BS-Seq data with coverage≥5, (b) includes SOLiD™ BS-Seq data with coverage≥10, (c) includes SOLiD™ BS-Seq data with coverage≥15 and (d) includes SOLiD™ BS-Seq data with coverage≥20. All of them show high correlation r≥0.94.

**
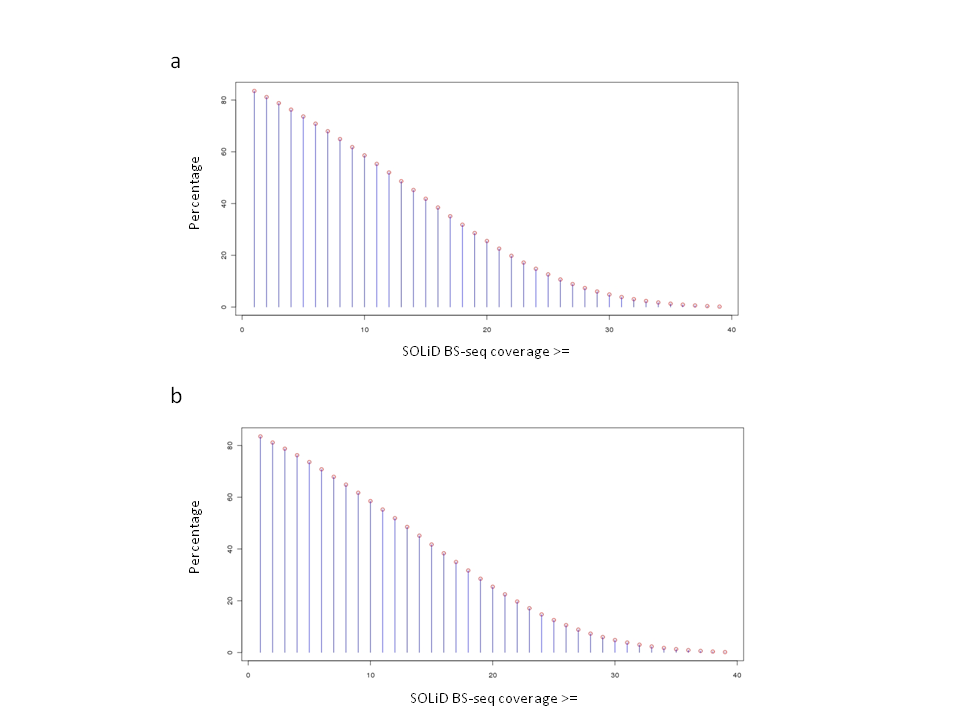
**

**Supplemental Figure S4: Coverage for SOLiD™ BS-seq.** Percentage of uniquely aligned SOLiD™ BS-Seq reads to the human reference (hg19/NCBI 37) for the forward (a) and the reverse (b) strand. Both strand coverages are equally distributed.

**Supplemental Figure S5: Genome-wide comparison of DNA methylation for different annotated repeat regions.** In total, 64.82% of all CpG sites within annotated repeat regions and 84.05% of all CpG sites outside of repeat regions were covered. Bar plots show distributions of DNA methylation levels within non-repeat regions, SINEs, LINEs, LTRs and Satellites. The y-axis indicates DNA methylation levels ((methylated reads)/(unmethylated reads + methylated reads)) assessed by SOLiD™ BS-seq.

MeanMethylation(Outside of repeat regions)=0.68

MeanMethylation(SINEs)=0.79, MeanMethylation(LINEs)=0.66

MeanMethylation(LTRs)=0.64, MeanMethylation(Satellites)=0.63

DNA methylation patterns on the forward and reverse strand were comparably established (Pearson r=0.90).

**
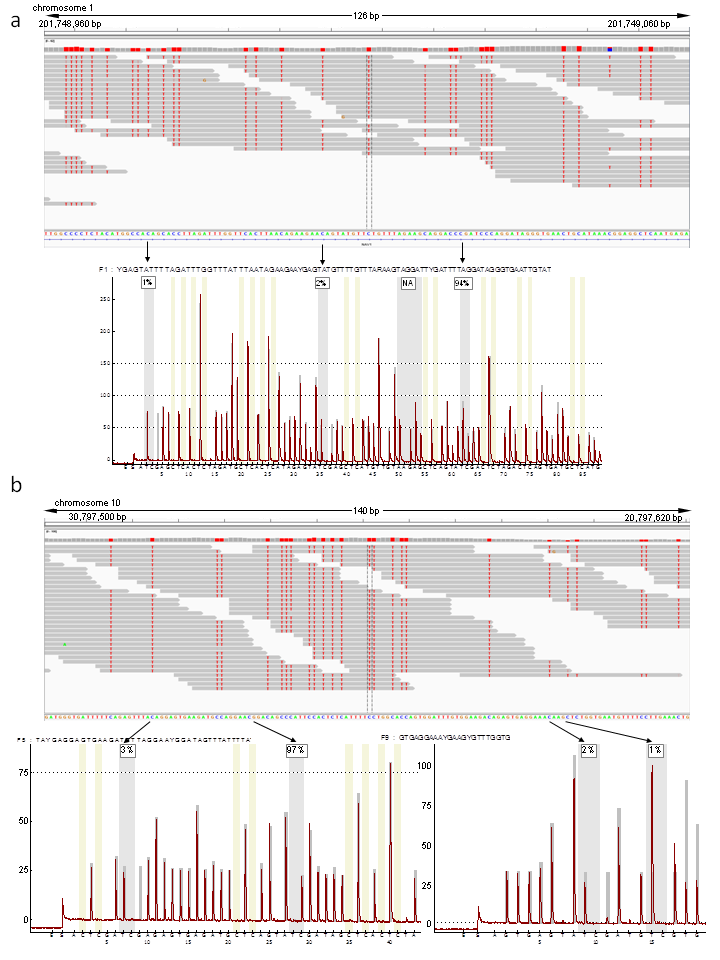
Supplemental Figure S6: Comparison of hallmark non-CpG sites showing significant non-CpG methylation in ESC cells^15^ analyzed by BS pyrosequencing and methylome sequencing.** No significant non-CpG methylation could be identified for hallmark sites on chromosome 1 (a) and chromosome 10 (b). For each region above the IGV plot of the bisulfite methylome data and below the BS pyrosequencing data are shown. The results of pyrosequencing are indicated as percentiles above each potentially methylated position (colored in grey). In each region one CpG site was analyzed which showed near to complete methylation.

**Supplemental Figure S7: Correlation analysis of significantly methylated non-CpG sites and transcriptional levels within RefSeq genes in DAUDI cells.** RefSeq genes were clustered (x=0, 0<x≤0.005, 0.005<x≤0.011, 0.011<x≤0.018) by their fraction of significantly methylated non-CpG sites related to the number of referenced non-CpGs within the respective RefSeq gene. The y-axis depicts transcriptional levels measured by FPKM values. A modest positive correlation between significantly methylated non-CpG sites and respective transcriptional levels can be observed. Similar results could be observed for transcriptional levels assessed by the Affymterix U133A chip (p_(x=0, 0.011≤x≤0.018)_=0.005, p_(0<x≤0.005, 0.011≤x≤0.018)_=0.004, p_(0.005<x≤0.011, 0.011≤x≤0.018)_=0.15).

**
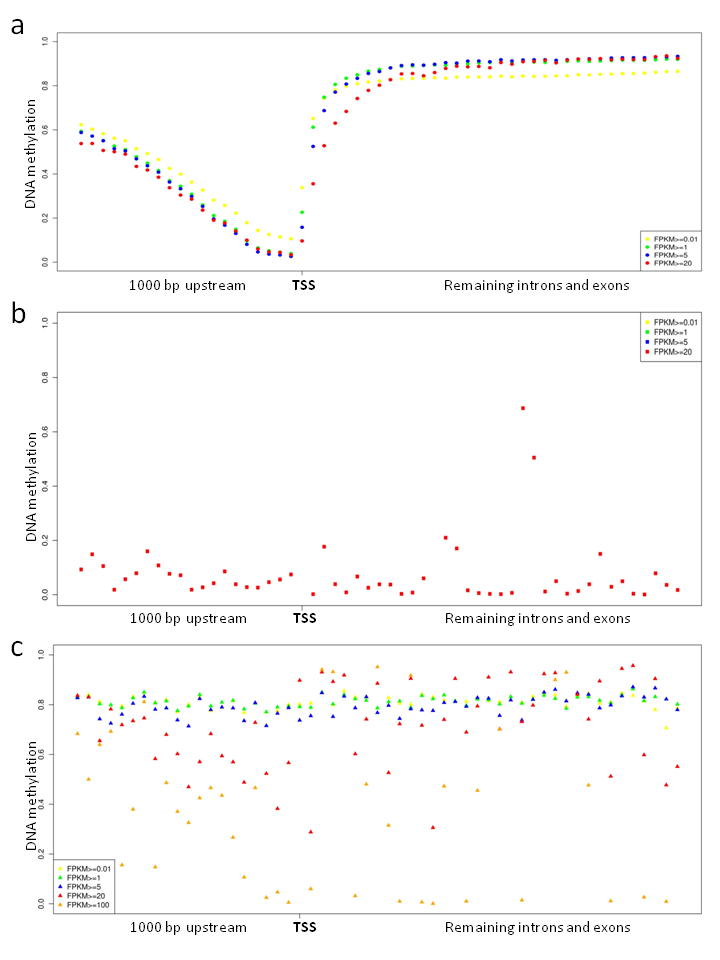
**

**Supplemental Figure S8: Methylation-expression correlation analyses.** CpG Methylation levels were averaged for annotated RefSeq gene regions and transcripts are clustered by their expression level in present and absent calls. A strong dependency of the location of CpGs related to their distance to the TSS and the transcript expression level can be observed for human data (a). Regarding the mitochondria (b), we observed an overall low DNA methylation degree independent from their expression levels. However, EBV (c) DNA methylation does not correlate within transcripts until an expression level of FPKM≥15.

**REFERENCES**

1. Nadkarni JS, Nadkarni JJ, Clifford P, Manolov G, Fenyö EM, Klein E. Characteristics of new cell lines derived from Burkitt lymphomas. *Cancer* 1969; **23:** 64 - 79.
2. Bormann Chung CA, Boyd VL, McKernan KJ, Fu Y, Monighetti C, Peckham HE. Whole methylome analysis by ultra-deep sequencing using two-base encoding. *PloS ONE* 2010; **5:** e9320.
3. Ranade SS, Bormann Chung C, Zon G, Boyd VL. Preparation of genome-wide DNA fragment libraries using bisulfite in polyacrylamide gel electrophoresis slices with formamide denaturation and quality control for massively parallel sequencing by oligonucleotide ligation and detection. *Anal Biochem* 2009; **390:** 126 - 135.
4. Kreck B, Marnellos G, Richter J, Krueger F, Siebert R, Franke A. B-SOLANA: an approach for the analysis of two-base encoding bisulfite sequencing data. *Bioinformatics* 2012; **28:** 428 - 429.
5. Krueger F, Kreck B, Franke A, Andrews SR. DNA methylome analysis using short bisulfite sequencing data. *Nat Methods*. 2012; **9**: 145 - 151.
6. Krueger F, Andrews SR. Bismark: a flexible aligner and methylation caller for Bisulfite-Seq applications. *Bioinformatics* 2011; **27:** 1571 - 1572.
7. Klostermeier UC, Barann M, Wittig M, Häsler R, Franke A, Gavrilova O, et al. A tissue-specific landscape of sense/antisense transcription in the mouse intestine. *BMC Genomics* 2011; **12:** 305.
8. Trapnell C, Pachter L, Salzberg SL. Tophat: discovering splice junctions with RNA-Seq. *Bioinformatics* 2009; **25:** 1105 - 1111.
9. Gentleman RC, Carey VJ, Bates DM, Bolstad B, Dettling M, Dudoit S, *et al.* Bioconductor: open software development for computational biology and bioinformatics. *Genome Biol* 2004; **5:** R80. Epub 2004 Sep 15.
10. Leenman EE, Panzer-Grümayer RE, Fischer S, Leitch HA, Horsman DE, Lion T, *et al.* Rapid determination of Epstein-Barr virus latent or lytic infection in single human cells using in situ hybridization. *Mod Pathol*. 2004; **17:** 1564 - 1572.
11. Li H, Durbin R. Fast and accurate short read alignment with Burrows-Wheeler transform. *Bioinformatics* 2009; **25:** 1754 - 1760.
12. Li H, Handsaker B, Wysoker A, Fennell T, Ruan J, Homer N, *et al.* The sequence alignment/map format and SAMtools. *Bioinformatics* 2009; **25:** 2078 - 2079.
13. DePristo MA, Banks E, Poplin R, Garimella KV, Maguire JR, Hartl C, *et al.* A framework for variation discovery and genotyping using next-generation DNA sequencing data. *Nat Genet* 2011; **43:** 491 - 498.
14. Wang K, Li M, Hakonarson H. ANNOVAR: functional annotation of genetic variants from high-throughput sequencing data. *Nucleic Acids Res* 2010; 38: e164. Epub 2010 Jul 3.
15. Lister R, Pelizzola M, Dowen RH, Hawkins RD, Hon G, Tonti-Filippini J, *et al.* Human DNA methylomes at base resolution show widespread epigenomic differences. *Nature* 2009; **462:** 315 - 322.
16. Lamprecht B, Walter K, Kreher S, Kumar R, Hummel M, Lenze D, *et al.* Derepression of an endogenous long terminal repeat activates the CSF1R proto-oncogene in human lymphoma. *Nat Med* 2010; **16:** 571 - 579, 1p following 579.
17. Karimi M, Johansson S ET. Using LUMA: a Luminometric-Based Assay for Global DNA-Methylation. *Epigenetics* 2006; **1:** 45 - 48.
18. Li Y, Vinckenbosch N, Tian G, Huerta-Sanchez E, Jiang T, Jiang H, *et al.* Resequencing of 200 human exomes identifies an excess of low-frequency non-synonymous coding variants. *Nat Genet* 2010; **42:** 969 - 972.
19. Ng SB, Turner EH, Robertson PD, Flygare SD, Bigham AW, Lee C, *et al.* Targeted capture and massively parallel sequencing of 12 human exomes. *Nature* 2009; **461:** 272 - 276.
20. Martín-Subero JI, Kreuz M, Bibikova M, Bentink S, Ammerpohl O, Wickham-Garcia E, *et al*. New insights into the biology and origin of mature aggressive B-cell lymphomas by combined epigenomic, genomic, and transcriptional profiling. *Blood* 2009; **113**: 2488 - 2497.
21. Hansen KD, Timp W, Bravo HC, Sabunciyan S, Langmead B, McDonald OG, *et al.* Increased methylation variation in epigenetic domains across cancer types. *Nat Genet* 2011; **43**:768-75.
22. Li Y, Zhu J, Tian G, Li N, Li Q, Ye M, *et al.* The DNA methylome of human peripheral blood mononuclear cells. *PLoS Biol* 2010; **8**:e1000533
